# Hypoxia and *P. gingivalis* Synergistically Induce HIF-1 and NF-*κ*B Activation in PDL Cells and Periodontal Diseases

**DOI:** 10.1155/2015/438085

**Published:** 2015-03-15

**Authors:** L. Gölz, S. Memmert, B. Rath-Deschner, A. Jäger, T. Appel, G. Baumgarten, W. Götz, S. Frede

**Affiliations:** ^1^Department of Orthodontics, Dental Clinic, University Hospital of Bonn, Welschnonnenstraße 17, 53111 Bonn, Germany; ^2^Center of Dento-Maxillo-Facial Medicine, University Hospital of Bonn, Sigmund-Freud-Straße 25, 53105 Bonn, Germany; ^3^Clinic of Anesthesiology and Intensive Care Medicine, University Hospital of Bonn, Welschnonnenstraße 17, 53111 Bonn, Germany

## Abstract

Periodontitis is characterized by deep periodontal pockets favoring the proliferation of anaerobic bacteria like *Porphyromonas gingivalis* (*P. gingivalis*), a periodontal pathogen frequently observed in patients suffering from periodontal inflammation. Therefore, the aim of the present study was to investigate the signaling pathways activated by lipopolysaccharide (LPS) of *P. gingivalis* (LPS-PG) and hypoxia in periodontal ligament (PDL) cells. The relevant transcription factors nuclear factor-kappa B (NF-*κ*B) and hypoxia inducible factor-1 (HIF-1) were determined. In addition, we analyzed the expression of interleukin- (IL-) 1*β*, matrix metalloproteinase-1 (MMP-1), and vascular endothelial growth factor (VEGF) in PDL cells on mRNA and protein level. This was accomplished by immunohistochemistry of healthy and inflamed periodontal tissues. We detected time-dependent additive effects of LPS-PG and hypoxia on NF-*κ*B and HIF-1*α* activation in PDL cells followed by an upregulation of IL-1*β*, MMP-1, and VEGF expression. Immunohistochemistry performed on tissue samples of gingivitis and periodontitis displayed an increase of NF-*κ*B, HIF-1, and VEGF immunoreactivity in accordance with disease progression validating the importance of the *in vitro* results. To conclude, the present study underlines the significance of NF-*κ*B and HIF-1*α* and their target genes VEGF, IL-1*β*, and MMP-1 in *P. gingivalis* and hypoxia induced periodontal inflammatory processes.

## 1. Introduction

According to recent studies the prevalence of periodontal diseases has increased ranging between 50 and 70% in adults over 30 years [1, 2]. Gingivitis and periodontitis are the most common forms of these oral diseases. Whereas gingivitis is reversible and restricted to superficial periodontal structures, periodontitis affects also deeper tissue layers leading to irreversible destructions which favor tooth loss. Therefore, the periodontal inflammation is the main cause of tooth loss in adults. Beside the esthetical and economical aspects, periodontal diseases are associated with a variety of systemic disorders. Periodontitis is under suspicion to increase the risk of coronary heart diseases, cerebral ischemia, and cancer [3–7]. Extrinsic factors like smoking as well as specific periodontal pathogens and the host immune system are the predominant etiological factors contributing to the onset and progression of periodontitis. Genetic variations which influence inflammatory and immunological reactions or the amount of matrix degeneration via matrix metalloproteinases (MMPs) seem to play a pivotal role in disease aggravation [8]. Periodontal pathogen bacteria activate several inflammatory and immunological pathways in local tissue cells and immune cells like monocytes and neutrophils. They have been divided into different complexes according to their pathogenicity and interdependent clustering [9].* Porphyromonas gingivalis* (*P. gingivalis*) belongs to the red complex which comprises bacteria of high pathogenicity.* P. gingivalis* is a gram-negative anaerobic pathogen which is often found in patients suffering from periodontitis. Current investigations have demonstrated that this pathogen is also correlated with atherosclerosis-associated systemic diseases supporting the hypothesis of* P. gingivalis* as an intermediate player [3, 6].

Lipopolysaccharide (LPS), a component of the outer membrane of gram-negative bacteria, is an important virulence factor of* P. gingivalis* activating the immune system via transmembrane toll-like receptors (TLRs), specific pattern recognition receptors (PRR). Ten different human TLRs (TLR1-10) have been characterized so far of which TLR4 is the founding member [10–12]. LPS from* Escherichia coli* (*E. coli*), the most abundant gram-negative bacteria of the gut, is the best studied ligand of TLR4, whereas LPS derived from* P. gingivalis* (LPS-PG) functions via toll-like receptor 2 (TLR2) and only in part via TLR4. Moreover, it has been shown that LPS-PG can also selectively inhibit TLR4 signaling depending on the exposed cell type [13, 14]. However, both TLR signaling pathways induce a cascade of molecules finally culminating in the activation of transcription factors like nuclear factor-kappa B (NF-*κ*B). NF-*κ*B is a transcription factor known to be responsible for cell proliferation, differentiation, apoptosis, and immune response [15, 16]. TLR signaling induces the phosphorylation of the inhibitor of NF-*κ*B (I*κ*B) followed by NF-*κ*B nuclear translocation. Consecutively, NF-*κ*B target genes like proinflammatory cytokines, for example, IL-1*β*, chemokines, adhesion molecules, and enzymes like MMPs, are expressed in order to recruit immune cells which minimize the growth and dissemination of bacteria. However, the overproduction of these mediators may also favor the induction and progression of inflammation worsening the local reaction. Therefore, an imbalance of the immune system (hypo- or hyper-reaction) may contribute to periodontal diseases.

At the same time, the inflammation in periodontal pockets is characterized by a reduced oxygen level and an acidic environment stabilizing the inflammatory state. Beside the inflammation-induced hypoxic environment also mechanical stress caused by chewing, grinding, or orthodontic treatment may provoke a deformation of blood vessels favoring ischemia. Previous investigations analyzing the effect of hypoxia have shown that reduced oxygen levels (1%) enhance the formation of reactive oxygen species (ROS) leading to oxidative stress in periodontal ligament cells [17]. The effect was even worse when the bacterial inflammation occurred in combination with a hypoxic environment. Hypoxia and proinflammatory cytokines like IL-1*β* are known to elicit an accumulation and activation of the transcription factor hypoxia inducible factor-1 (HIF-1) [18–20]. HIF-1 is a heterodimeric transcription factor consisting of two subunits: HIF-1*α* and HIF-1*β*. HIF-1*α* is located in the cytosol and predominantly regulated by the cellular oxygen tension. Under normoxic conditions prolyl hydroxylases (PHDs) or asparagine hydroxylases hydroxylate HIF-1*α* on specific proline and asparagine residues initiating the proteasomal degradation of the protein. In contrast, HIF-1*β* is constitutively expressed and located in the nucleus. Under hypoxic conditions PHDs are inhibited leading to the stabilization of HIF-1*α*. Consecutively, HIF-1*α* translocates into the nucleus, dimerizes with HIF-1*β* and followed by the transcription of various genes like vascular growth factor (VEGF) involved in short-term and long-term adaptations to hypoxia including upregulation of glycolysis, angiogenesis, and immune cell recruitment [21]. Moreover, recent studies have demonstrated an association between NF-*κ*B and HIF signaling with NF-*κ*B playing a crucial role in basal and stimulated HIF-1*α* mRNA expression [19, 20, 22].

The importance of bacterial and oxidative stress in oral diseases has been shown recently [17]. However, the interplay between NF-*κ*B and HIF as well as the mechanism of hypoxia and bacterial inflammation in the periodontium still needs to be determined. This is of clinical significance as bacterial inflammation is naturally accompanied by a hypoxic microenvironment and both effects are highly connected favoring the aggravation of periodontal inflammation and associated systemic diseases. The aim of the following study was, therefore, to evaluate the impact of both transcription factors and their inflammation-associated target genes during bacterial and hypoxic stress. We hypothesized that the combination of both stimuli disturbs the periodontal homeostasis leading to a progression of periodontal diseases as a consequence of the activation of NF-*κ*B and HIF-1*α*.

## 2. Materials and Methods

### 2.1. Isolation and Characterization of Periodontal Ligament Cells

Human periodontal ligament (PDL) fibroblasts were harvested from caries-free and periodontally healthy teeth, which had to be extracted due to orthodontic reasons. The approval of the Ethics Committee of the University of Bonn (Lfd. Nr. 086/11) and parental and patients' consent were obtained. Cells were collected as previously described [17, 23]. In brief, PDL cells were harvested from the medial part of the tooth root to avoid contamination by cells derived from the gingiva and the dental germ. Using explant technique PDL cells were cultured in Dulbecco's minimal essential medium (DMEM, Invitrogen, Karlsruhe, Germany) supplemented with 10% fetal bovine serum (FBS, Invitrogen) 100 units/mL penicillin and 100 mg/mL streptomycin (Biochrom, Berlin, Germany) at 37°C in a humidified atmosphere of 5% CO_2_. Cells from nine different individuals were characterized in the 3rd passage by the analysis of the known PDL markers alkaline phosphatase (ALP), osteocalcin (OC), osteopontin (OP), periostin, S100 A4 [23]. After phenotyping cells were pooled and used for experiments in 5th passage.

### 2.2. Cell Experiments

Cells were seeded (50,000 cells/well) on six-well plates and grown to 80% confluence. Experiments were performed under normoxic (21% O_2_) or hypoxic conditions (1% O_2_) and 5% CO_2_ (INCO 153, O_2_/CO_2_ incubator; Memmert GmbH, Schwabach, Germany) for 2, 4, 8, 24, and 48 h. Ultrapure Lipopolysaccharide (LPS) from* Porphyromonas gingivalis* (LPS-PG) (Invivogen, San Diego, USA) was used to mimic inflammatory conditions* in vitro* as has been described before [17].

### 2.3. Immunofluorescence

In order to evaluate the intracellular signaling pathways involved in the induction of a hypoxic and inflammatory response, activation of the transcription factors HIF-1*α* and NF-*κ*B was determined using immunofluorescence. For this purpose cells were cultured on coverslips to 60% confluence. Subsequently, PDL cells were incubated under normoxic or hypoxic conditions in the presence or absence of LPS-PG. Afterwards, medium was removed; cells were washed three times with PBS and fixed with 250 mL cooled methanol/aceton (1 : 1) at −20°C for 10 min. The solution was removed and cells remained at room temperature for 10 min to dry. After blocking with 3% BSA-PBS for 10 min, cells were incubated with antibodies (Ab) directed against HIF-1*α* and NF-*κ*B (Novus Biologicals, Littleton CO, USA; Santa Cruz Biotechnologies, Heidelberg, Germany) 1 : 50 in PBS and 3% BSA for 2 h. Cells were washed with PBS before the species-specific secondary Ab conjugated to FITC (dilution 1 : 400 in PBS) were added for additional 90 min. Cells were washed again and covered on slides with DAPI containing mounting medium (Dako). The following day cells were scanned with the Zeiss Axio Imager A1 fluorescence microscope (HBO 100; Carl, Zeiss, Jena, Germany), an AxioCam MRc camera (Carl, Zeiss), and the AxioVision 4.7 software (Carl, Zeiss).

### 2.4. Analysis of Gene Expression

At the end of the experiments cells were lysed with 4M guanidinthiocyanat (GTC) (Roth, Karlsruhe, Germany) and total RNA was extracted using the acid guanidiniumthiocyanate/phenol/chloroform extraction method [24]. A total of 1 *μ*g of RNA were reverse transcribed into cDNA by use of 200 U Maloney Murine Leukemia Virus (MMLV) reverse transcriptase (Bio-Rad, Munich, Germany). cDNA expression was detected by real-time PCR (RT-PCR) using specific primers as listed in Table 1 and SYBR Green as fluorescent dye on the VIIA7 detection system (Applied Biosystems, Darmstadt, Germany). Two *μ*L of cDNA served as template in a 20 *μ*L reaction mixture containing 10 *μ*L QuantiTect SYBR Green Master Mix (Qiagen, Hilden, Germany). The mixture was heated initially to 50°C for 2 min, 95°C for 15 min, and then followed by 40 cycles with denaturation at 95°C for 15 s, annealing and extension at 60°C for 1 min. Amounts of specific cDNA were normalized to the housekeeping gene 18S and cDNA expression was calculated as relative expression to the respective control (ΔΔct method).

### 2.5. Analysis of Protein Expression

In order to determine the release of specific mediators, cell free supernatants were collected at the end of the respective incubation periods before the addition of GTC. Supernatants were transferred to new tubes and kept at −80°C until further analysis. For the measurement of MMP-1 and VEGF protein the Quantikine enzyme-linked immunoabsorbent assay (ELISA) system was used according to the manufacturer's instruction (R&D Systems, Wiesbaden, Germany).

### 2.6. Histology and Immunostaining

Periodontal tissues were obtained before periodontal therapy and after the approval of the Ethics Committee of the University of Bonn and parental as well as patients' written consent. Tissues were fixed in 4% phosphate-buffered paraformaldehyde for 24 h, followed by hydration and dehydration in an ascending ethanol series which ended in paraffin embedding. For further investigations referring to NF-*κ*B, HIF-1*α*, and VEGF expression in healthy and inflamed periodontium, immunohistochemistry (IHC) was performed on 2-*μ*m-thick paraffin-embedded sections using the peroxidase-conjugated avidin-biotin method. For this purpose sections were deparaffinized and rehydrated, rinsed with tris-hydroxymethylaminomethane-buffered saline solution (TBS) at pH 7.4 for 10 min, and then soaked in 70 mL methanol containing 700 *μ*L of 30% H_2_O_2_ for 45 min in the dark to block endogenous peroxidase activity. Afterwards, tissue sections were rinsed and preincubated with TBS-bovine serum albumin (TBS/BSA 4%) for 20 min to avoid unspecific background staining. Then, sections were incubated with primary antibodies directed against NF-*κ*B (Santa Cruz, Biotechnologies, Heidelberg, Germany), HIF-1*α* (Novus Biologicals, Littleton CO, USA), and VEGF (R&D systems, Wiesbaden, Germany) in a 1 : 100 working dilution in TBS/BSA at 4°C overnight in a humidified chamber. Subsequently, sections were washed again in TBS and incubated with a peroxidase-labeled polymer conjugated to a goat anti-rabbit immunoglobulin (Envision; Dako A/S, Glostrup, Denmark) for 30 min in a humidified chamber at room temperature. Antibody complexes were visualized using diaminobenzidine for 10 min resulting in brown staining. Thereafter, the slides were rinsed, counterstained with Mayer's haematoxylin, rinsed again, and cover slipped. Negative controls were prepared by omission of the primary antibodies, as well as both the primary or secondary antibody from the staining procedures using TBS/BSA instead. Afterwards, stained tissue sections were analyzed using the Zeiss AxioScop 2 microscope (Carl, Zeiss, Jena, Germany) with an AxioCam MRc camera (Carl, Zeiss) and the AxioVision 4.7 software (Carl, Zeiss).

### 2.7. Statistical Analysis

All experiments were performed in triplicate and mRNA quantification are given as means ± SD. Time-matched untreated cells incubated under normoxic conditions served as controls. Results were compared by ANOVA; Dunnett's and Tukey-Kramer tests were performed as posttests to calculate whether differences between the respective control (time-matched) or treated groups were significant. A *P* value of 0.05 was considered to be significant.

## 3. Results

### 3.1. Effects of LPS-PG and Hypoxia on IL-1*β* and MMP-1 Expression in PDL Cells

To evaluate the effects of bacterial inflammation and hypoxia on IL-1*β* and MMP-1 expression in human periodontal ligament fibroblasts, cells were incubated with the periodontal pathogen LPS-PG in the presence or absence of hypoxia for different time periods.

#### 3.1.1. IL-1*β*

**Figure 1:**
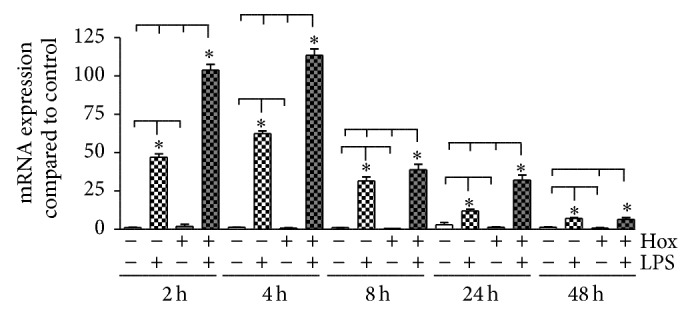
*Induction of IL-1*β* mRNA in PDL cells*. PDL cells were cultured under normoxic or hypoxic condition and stimulated with LPS-PG (1 *μ*g/mL). Cells cultured in normoxia without LPS stimulation served as control. IL-1*β* mRNA expression was analyzed by real-time PCR after 2, 4, 8, 24, and 48 h. Statistical differences were analyzed by one-way ANOVA and Dunnett's test as well as Tukey's multiple comparison test; ^*^ *P* < 0.05 indicates a significant difference between the groups (means ± SD; *n* = 9).

LPS-PG induced a significant increase in IL-1*β* gene expression almost 2 h after the onset of stimulation compared to the untreated control (48-fold; *P* < 0.001; Figure 1). The effect was even more prominent after 4 h, declined afterwards but was still present after 48 h (*P* < 0.01). Hypoxia alone elicited no significant changes concerning IL-1*β* mRNA expression. However, the hypoxic condition nearly doubled the effect of LPS-PG after 2 h and even after 4 h (100-fold compared to control; *P* < 0.001; Figure 1). To test whether the increase in IL-1*β* mRNA expression was followed by the release of the protein, cell culture supernatant was collected after each time point. We did not find any significant effects concerning the release of the proinflammatory cytokine independent of the treatment (*P* > 0.05; data not shown).

#### 3.1.2. MMP-1

**Figure 2:**
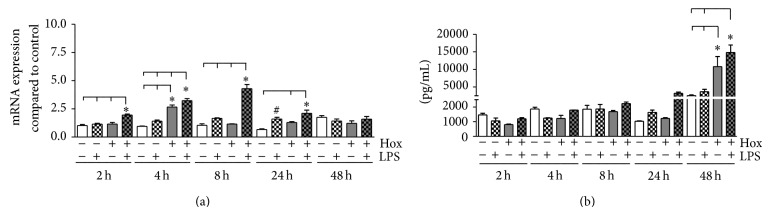
*Expression of MMP-1 in PDL cells*. (a) PDL cells were cultured under normoxic or hypoxic condition and stimulated with LPS-PG (1 *μ*g/mL). Untreated cells cultured in normoxia served as control. MMP-1 mRNA expression was quantitated by real-time PCR after 2, 4, 8, 24, and 48 h. Statistical differences were analyzed by one-way ANOVA followed by different post hoc tests (Dunnett's and Tukey's multiple comparison test); a *P* value < 0.05 was assumed as significant. ^#^ *P* < 0.05 depicts a significant difference with respect to the time-matched control, ^*^ *P* < 0.05 indicates a significant difference between groups (means ± SD; *n* = 9). (b) MMP-1 protein secretion by PDL fibroblasts was determined using the Quantikine enzyme-linked immunoabsorbent assay (ELISA) system according to the manufacturer's instruction. Supernatants of PDL cells stimulated with or without LPS-PG (1 *μ*g/mL) under normoxic or hypoxic condition were collected after varying time points (2, 4, 8, 24, and 48 h). Statistical differences were analyzed by one-way ANOVA followed by different post hoc tests (Dunnett's and Tukey's multiple comparison test); a *P* value < 0.05 was assumed as significant. ^*^ *P* < 0.05 indicates a significant difference between groups (means ± SD; *n* = 9).

In order to analyze the effects of LPS-PG and hypoxia on the release of matrix metalloproteinases by PDL cells, we measured the expression of MMP-1 (Figure 2). A significant enhancement of MMP-1 mRNA under hypoxic conditions alone was detected only after 4 h, while LPS-PG alone induced a slight but significant effect on MMP-1 expression exclusively after 24 h (Figure 2(a)). However, the combination of LPS-PG and hypoxia led to a significant induction of MMP-1 mRNA expression compared to all other treatments within the first 24 h of stimulation (*P* < 0.05). The upregulation of MMP-1 mRNA was followed by an enhanced but time-displaced release of MMP-1 protein (Figure 2(b)). At early time points (2, 4, and 8 h), neither LPS-PG nor hypoxia nor the combination of both elicited a significant alteration in MMP-1 protein concentrations in the supernatant (*P* > 0.05; Figure 2(b)). However, LPS-PG in combination with hypoxia markedly elevated MMP-1 protein after 24 h. Extending the incubation period to 48 h, hypoxic conditions alone as well as in combination with LPS-PG increased MMP-1 levels significantly (5-fold; *P* < 0.001; Figure 2(b)).

### 3.2. Activation of NF-*κ*B

The transcription factor complex NF-*κ*B is important for the activation of several genes involved in inflammatory processes. Therefore, we determined the activation of NF-*κ*B by means of immunofluorescence in PDL cells and by immunohistochemistry in tissue samples of healthy and inflamed gingiva and periodontium.

#### 3.2.1. Activation of NF-*κ*B in PDL Cells

**Figure 3:**
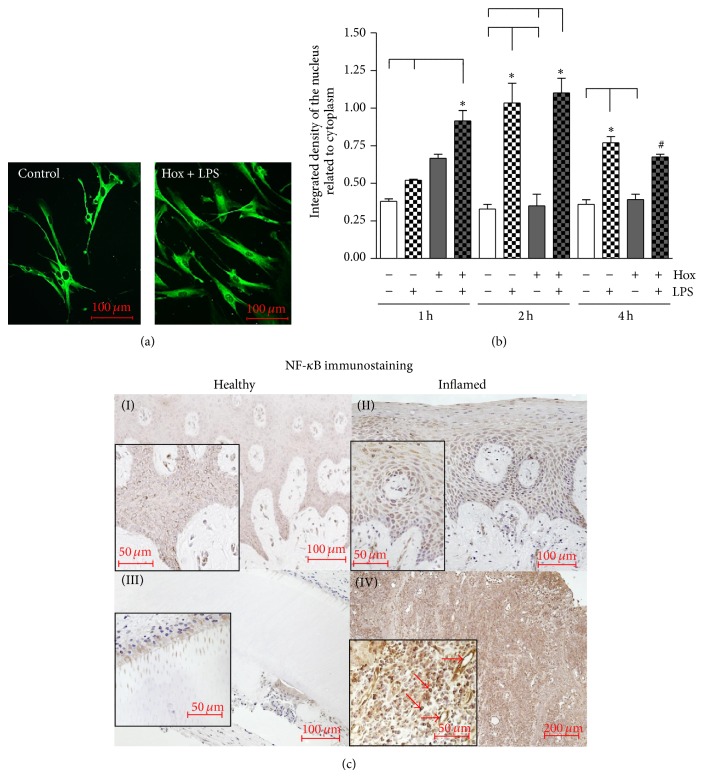
*Activation of NF-*κ*B*. (a) NF-*κ*B was visualized by immunofluorescence staining. PDL cells were cultured under normoxic (control) or hypoxic condition (Hox) and stimulated with LPS-PG (1 *μ*g/mL). NF-*κ*B activation was visible as enhanced nuclear staining. Representative pictures of control cells and a 1 hour LPS-PG treatment under hypoxic conditions are shown. The scale bars indicate 100 *μ*m. (b) Density of NF-*κ*B nuclear staining after 1, 2, and 4 h treatment was determined in relation to total cell area using the freely available image-processing software ImageJ 1.43 (http://rsb.info.nih.gov/ij). Statistical analysis of the processed immunofluorescence data were performed by one-way ANOVA followed by Dunnett's test and Tukey's multiple comparison test; a *P* value < 0.05 was assumed as significant. ^#^ *P* < 0.05 depicts a significant difference with respect to time-matched control, ^*^ *P* < 0.05 indicates a significant difference between groups (means ± SD; *n* = 6). (c) Tissue samples from healthy gingiva (I), gingivitis (II), healthy periodontal ligament (III), and periodontitis (IV) were obtained after approval of the Ethics Committee of the University of Bonn and parental as well as patients' written consent (*n* = 3). Polyclonal primary antibody raised against the p65 subunit of NF-*κ*B was used in a concentration of 1 : 100 for immunohistochemistry. We observed an increase in NF-*κ*B immunostaining in accordance to the progression of periodontal inflammation. Arrows indicate the nuclear staining of NF-*κ*B in endothelial cells as well as immune cells in tissue samples of periodontitis. The scale bars indicate 50, 100, or 200 *μ*m in dependence of the magnifications.

Under control conditions NF-*κ*B is located in the cytosol and no positive staining was observed in the nuclei. After stimulation of PDL cells with LPS-PG under hypoxic conditions a considerably staining of the nuclei was detected demonstrating the nuclear translocation and activation of NF-*κ*B (Figure 3(a)). Effects of LPS-PG and hypoxia on NF-*κ*B activation were evaluated at least after 1, 2, and 4 h incubation. Densitometrical analyses of the immunofluorescence pictures revealed an activation of NF-*κ*B after 1 h of LPS-PG stimulation which reached significance after 2 h (*P* < 0.001) and even after 4 h incubation under normoxic conditions (*P* < 0.01; Figure 3(b)). As shown in Figure 3(b), LPS-PG in combination with hypoxia provoked a distinct nuclear translocation of the transcription factor after 1 h, reached a maximum after 2 h and declined (*P* < 0.01). Only after 1 h incubation hypoxia enhanced the LPS-PG induced NF-kB activation significantly. Instead, hypoxia alone induced a slight but not significant increase of NF-*κ*B activation after 1 h which declined afterwards (*P* > 0.05).

#### 3.2.2. Activation of NF-*κ*B in Periodontal Tissues

Periodontal tissue sections (healthy gingiva and periodontal ligament (PDL), gingivitis, and periodontitis) were obtained from three different patients for each group and immunohistochemistry was performed (Figure 3(c)).

In the healthy gingiva a moderate staining of the epithelial (keratinocytes) and subepithelial cells (mainly fibroblasts) was observed (Figure 3(c)-(I)). Immunoreactivity for the transcription factor NF-*κ*B appeared primarily in the cytosol of these cells and was not restricted to any epithelial layer. Tissue sections of patients suffering from gingivitis revealed only a slight increase of NF-*κ*B immunostaining limited to the cytosol of keratinocytes, fibroblasts, and immune cells (Figure 3(c)-(II)).

In the healthy periodontium, predominantly PDL cells located near to the tooth root surfaces and cementocytes displayed a positive NF-*κ*B staining (Figure 3(c)-(III)). Moreover, we detected NF-*κ*B in dentin tubuli beside these cells. In inflamed periodontal tissue sections, we observed a strong and generalized immunoreactivity for NF-*κ*B in the cytosol of almost every cell type (Figure 3(c)-(IV)). Intensive nuclear localization of the transcription factor could be allocated to immune cells and endothelial cells (red arrows, Figure 3(c)-(IV)).

### 3.3. HIF Activation in PDL Cells and Periodontal Tissues

The transcription factor complex HIF-1 is the key regulator of cellular adaptation to hypoxic conditions and is known to be activated by several inflammatory mediators. Therefore, we tested the activation of HIF-1 in PDL cells after stimulation with LPS-PG and hypoxia. In addition, we tested for the accumulation of HIF-1*α* in human tissue samples of healthy and inflamed gingiva and periodontal ligament.

#### 3.3.1. HIF-1*α* Activation in PDL Cells

**Figure 4:**
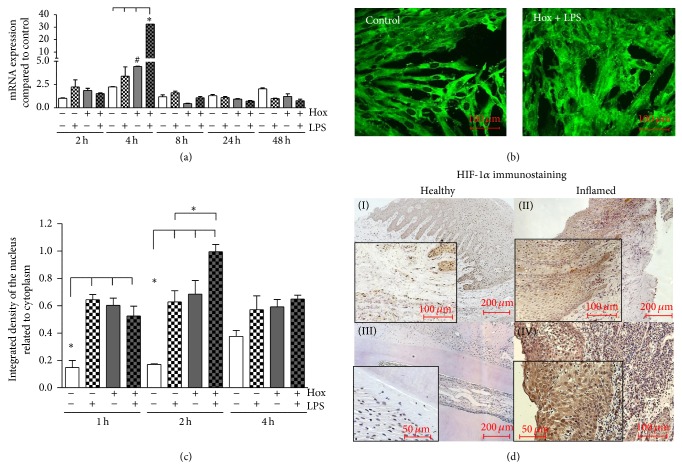
*Activation of HIF-1α*. (a) PDL cells were incubated for the indicated time periods with LPS-PG under normoxic or hypoxic conditions. HIF-1*α* mRNA expression was quantitated by real-time PCR, and expression levels were calculated as n-fold induction of the respective time matched untreated normoxic control. Statistical differences were analyzed by one-way ANOVA followed by different post hoc tests (Dunnett's and Tukey's multiple comparison test); ^#^ *P* < 0.05 depicts a significant difference with respect to time-matched control, ^*^ *P* < 0.05 indicates a significant difference between groups (means ± SD; *n* = 6). (b) PDL cells were cultured under normoxic (control) or hypoxic conditions (Hox) and stimulated with LPS-PG (1 *μ*g/mL). HIF-1*α* protein was visualized by immunofluorescence staining. Representative pictures of cells under normoxic control conditions and after a 2-hour LPS-PG treatment under hypoxic conditions are shown. The scale bars indicate 100 *μ*m. (c) PDL cells were incubated for 1, 2, and 4 hours. HIF-1*α* protein was detected by immunofluorescence. Density of nuclear HIF-1*α* staining was determined as in relation to total cell area using the freely available image-processing software ImageJ 1.43 (http://rsb.info.nih.gov/ij). Statistical analysis of the processed data were performed by one-way ANOVA followed by Tukey's multiple comparison test; ^*^ *P* < 0.05 indicates a significant difference between groups (means ± SD; *n* = 6). (d) Tissue samples from healthy gingiva (I), gingivitis (II), healthy periodontal ligament (III), and periodontitis (IV) were obtained after approval of the Ethics Committee of the University of Bonn and parental as well as patients' written consent (*n* = 3). Polyclonal primary antibody raised against HIF-1 was used in a concentration of 1 : 100 for immunohistochemistry. We observed an increase of HIF-1*α* immunostaining in accordance to the progression of periodontal inflammation. The scale bars indicate 50, 100, or 200 *μ*m in dependence of the magnifications.

As most other cell types PDL cells displayed a constitutive expression of HIF-1*α* mRNA. A significant increase in HIF-1*α* mRNA expression was observed only after 4 h incubation under hypoxic conditions and even more pronounced with an almost 30-fold increase after stimulation with LPS-PG under these conditions (*P* < 0.001; Figure 4(a)). For all other stimulation periods no effects on HIF-1*α* expression were detectable.

Since the abundance and activity of HIF-1 is predominantly regulated on the posttranslational level, we tested next whether LPS-PG and hypoxia induced the stabilization and nuclear translocation of the HIF-1*α* protein in PDL cells using immunofluorescence. As shown in Figure 4(b), HIF-1*α* protein was visible in the cytosol of PDL cells even under normoxic control conditions. Incubation with LPS-PG under hypoxic conditions caused a marked increase in nuclear staining for HIF-1*α* (Figure 4(b)). To get more detailed information about the time course of HIF-1 activation, we stimulated the cells for 1, 2, and 4 h with LPS-PG under normoxic and hypoxic conditions. To quantitate the changes in nuclear HIF-1*α* accumulation, densitometrical analyses of immunofluorescence pictures were performed. As expected, hypoxia alone caused a significant stabilization and nuclear translocation of HIF-1*α* (*P* < 0.01; Figure 4(c)). Stimulation with LPS-PG under normoxic conditions revealed a nuclear accumulation of HIF-1*α* likewise. The maximum in nuclear HIF-1*α* accumulation was observed after 2 h treatment with LPS-PG under hypoxic conditions (*P* < 0.001). Extending the stimulation period to 4 h caused no further increase in nuclear HIF-1*α*, but the protein in the nuclei was still elevated when compared to the untreated controls (Figure 4(c)).

#### 3.3.2. Activation of HIF-1*α* in Periodontal Tissues

Periodontal tissue sections (healthy gingiva and periodontal ligament (PDL), gingivitis, and periodontitis) were obtained from three different patients for each group and immunohistochemistry for HIF-1*α* was performed (Figure 4(d)).

In the healthy gingiva a weak HIF-1*α* staining was observed in gingival epithelial cells (keratinocytes) located close to the basal lamina and spatially distributed in subepithelial cells (primarily fibroblasts). Immunoreactivity for the transcription factor HIF-1*α* appeared in the cytosol and in the nuclei of these cells (Figure 4(d)-(I)). Tissue sections of patients suffering from gingivitis revealed a substantial increase of HIF-1*α* positive cells. Immunostaining was detectable in large areas of the gingival epithelium, in subepithelial fibroblasts, and possibly in infiltrating immune cells (Figure 4(d)-(II)).

In the healthy periodontium, no HIF-1*α* staining was visible (Figure 4(d)-(III)). In tissue samples of periodontitis, we observed a dramatic and generalized immunoreactivity for HIF-1*α* in the cytosol and the nuclei of almost every cell type (Figure 4(d)-(IV)). Additionally, the number of HIF-1*α* positive cells, most likely fibroblasts and infiltrating immune cells, strongly increased in the subepithelium.

### 3.4. Induction of VEGF

After demonstrating the activation of HIF by LPS-PG and hypoxia in PDL cells, we were interested whether the HIF target gene VEGF was consecutively upregulated.

#### 3.4.1. VEGF Release from PDL Cells

PDL cells were incubated up to 48 h with LPS-PG in the presence or absence of hypoxia. At the end of indicated incubation periods, cell culture supernatants were collected and VEGF concentration determined by ELISA.

**Figure 5:**
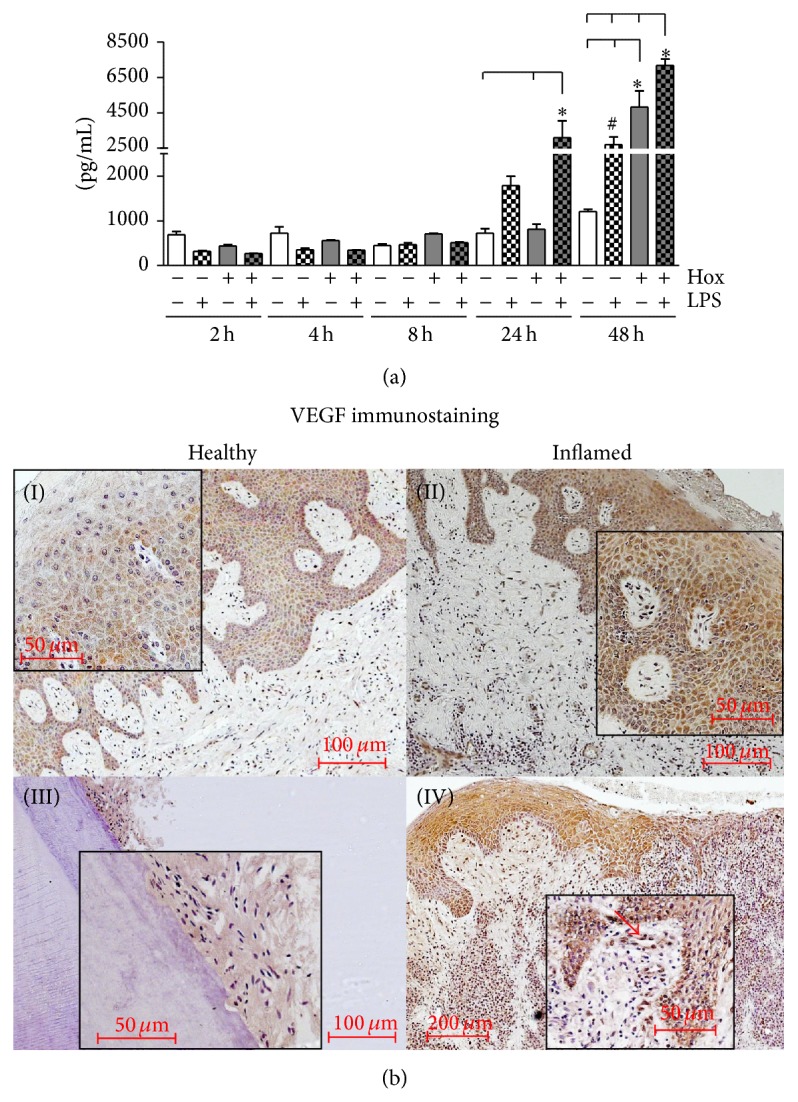
*VEGF protein expression*. (a) VEGF protein expression in PDL cells was measured using ELISA. Supernatants of PDL cells, stimulated with or without LPS-PG (1 *μ*g/mL) under normoxic or hypoxic condition, were collected after varying time points (2, 4, 8, 24, and 48 h). Statistical differences were analyzed by one-way ANOVA and the post hoc Dunnett's and Tukey's multiple comparison test; ^#^ *P* < 0.05 depicts a significant difference with respect to the time-matched control, ^*^ *P* < 0.05 indicates a significant difference between groups (means ± SD; *n* = 9). (b) Healthy gingiva (I), gingivitis (II), healthy periodontal ligament (III), and periodontitis (IV) were obtained after the approval of the Ethics Committee of the University of Bonn and parental as well as patients' written consent (*n* = 3). A polyclonal primary antibody against VEGF was used in a concentration of 1 : 100 for immunohistochemistry. The scale bars indicate 50, 100, or 200 *μ*m in dependence of the magnifications.

As depicted in Figure 5(a), PDL cells constitutively release small amounts of VEGF protein. Nevertheless, detectable changes in the amount of VEGF protein induced by LPS-PG were seen earliest after 24 h and reached significance after 48 h (*P* < 0.05 compared to the time-matched control). Hypoxia alone caused a significant increase in VEGF protein also after 48 h in comparison to control and LPS-PG treatment under normoxic conditions (*P* < 0.05). Hypoxia in combination with LPS-PG enhanced the LPS-PG induced release of VEGF significantly after 24 h and 48 h (*P* < 0.001; Figure 5(a)).

#### 3.4.2. Implication of VEGF in Periodontal Tissues

Periodontal tissue sections (healthy gingiva and periodontal ligament (PDL), gingivitis, and periodontitis) were obtained from three different patients for each group and immunohistochemistry for VEGF was performed.

In healthy gingival tissue samples, we observed a weak but distinct immunostaining for VEGF primarily located in the cytosol of keratinocytes (Figure 5(b)-(I)). In the subepithelium no prominent signs of VEGF immunoreactivity were visible. In contrast, in inflamed gingival tissue sections (gingivitis) VEGF was enhanced in keratinocytes. In addition, we detected VEGF staining in endothelial cells of the subepithelium and also in some infiltrating immune cells. Moreover, VEGF in the intercellular space was visible (Figure 5(b)-(II)). In the healthy periodontal ligament VEGF immunoreactivity was very weak and limited to the desmodontium, predominantly located in the cytosol and surrounding of PDL fibroblasts (Figure 5(b)-(III)). Instead, in tissue samples of patients suffering from periodontitis, strong immunoreactivity was found in keratinocytes as well as in endothelial cells of the subepithelial layer (Figure 5(b)-(IV), red arrow) and infiltrating immune cells. The amount of positively stained infiltrating cells was higher in periodontitis samples than in samples from gingivitis according to the progression of inflammation.

## 4. Discussion

Periodontal pathologies have become of major concern in the last decades due to the increasing prevalence and association with systemic diseases [1–4, 25]. Periodontitis is the most common form of these pathologies and in contrast to gingivitis not restricted to superficial epithelial layers. Extrinsic factors like smoking as well as periodontal pathogen bacteria and the host immune response play a crucial role in the etiology and progression of this oral inflammation.* P. gingivalis* is known to be involved in the onset and aggravation of periodontitis but is also implicated in the initiation and progression of systemic nonoral diseases like atherosclerosis or cancer [3, 4, 7]. Its virulence factor LPS activates the immune system via TLRs leading to an inflammatory response with increased expression of proinflammatory cytokines as well as matrix metalloproteinases favoring the recruitment of immune cells like monocytes/macrophages and neutrophils [13, 14, 26]. Moreover, periodontal inflammation is suspected to aggravate local tissue hypoxia in the deep periodontal pockets [27, 28]. Direct measurements of the oxygen saturation (SO_2_) in periodontal tissues revealed a distinct lowered SO_2_ in periodontitis patients compared to healthy volunteers (71% SO_2_ versus 77% SO_2_; preliminary data provided by Professor Dr. W. Götz). In addition to this inflammation-induced hypoxic microenvironment, mechanical stress as a consequence of chewing, grinding, or orthodontic treatment may contribute to a reduced oxygen supply caused by deformation and compression of small blood vessels. We therefore investigated how LPS-PG in combination with hypoxia modulates the expression of proinflammatory cytokines, matrix metalloproteinases, and VEGF in periodontal ligament cells.

At first, we could demonstrate that LPS-PG and hypoxia induced a significant increase in IL-1*β* gene expression. While hypoxia alone exerts no measurable effects, the LPS-induced upregulation of IL-1*β* expression was significantly enhanced by hypoxic conditions. These results were in line with very recently published work from Jian and coworkers, who described an enhancement of LPS-induced inflammatory cytokine expression in isolated PDL cells by hypoxia [29]. Unexpectedly, a significant increase in IL-1*β* protein release was not measurable in our experimental setting. It is generally accepted that the release of IL-1*β* is a multistep process starting with the upregulation of gene expression, followed by the production of an inactive pro-IL-1*β* which is cleaved by caspase 1 to the active form after activation of the inflammasome [30]. For that reason it is conceivable that additional inflammatory or environmental stimuli are necessary to induce the release of IL-1*β* by PDL cells.

Apart from proinflammatory cytokines and reactive oxygen species, matrix metalloproteinases released by various cell types are discussed to be involved in the progression of periodontal diseases [17, 31–33]. Several polymorphisms in the MMP-1 gene resulting in a constitutive active form of the proteinase were shown to coincide with severe forms of periodontitis [34]. Kraus and coworkers demonstrated an upregulation of MMP-1 after stimulation with LPS-PG in gingival cells [35]. Very recently these observations were confirmed by Kong et al. [36]. The authors observed an upregulation (3-fold) of MMP-1 mRNA expression after 24 h stimulation with LPS-PG and a weak but significant increase in secreted protein. Incubating PDL cells with LPS-PG under normoxic conditions revealed an induction of MMP-1 mRNA after 24 h. In combination with hypoxia the maximum of LPS-PG induced MMP-1 expression shifted to earlier time points. In 2012 Song and coworkers demonstrated the enhancement of matrix metalloproteinases, especially MMP-2 and the respective tissue inhibitors TIMP-1 and TIMP-2 after hypoxic stimulation of PDL cells [37]. In the present study hypoxia alone caused an upregulation of MMP-1 mRNA only after 4 hours. From these data we conclude that MMP-1 secreted by PDL cells contributes to the tissue destruction seen in periodontitis and gingivitis and that hypoxia further aggravates the effects of LPS-PG on MMP-1 expression.

In order to elucidate the signaling pathways contributing to the inflammatory and hypoxic induction of IL-1*β* and MMP-1, we analyzed the activation patterns of the transcription factor complexes NF-*κ*B and HIF. NF-*κ*B was shown to be activated by a broad variety of inflammatory stimuli and NF-*κ*B target genes are involved in the induction and progression of inflammation, necrosis, apoptosis, and malignant cell proliferation [38, 39]. In addition, Winning et al. demonstrated the activation of NF-*κ*B by severe hypoxia in monocytic cells [40]. In line with these observations, PDL cells displayed a moderate transient upregulation of NF-*κ*B with a maximum after one hour when incubated under hypoxic conditions without additional LPS-PG. The loss of hydroxylation of IKK*β*, an upstream kinase within the NF-*κ*B signaling cascade, was identified to be in part responsible for the hypoxic activation of NF-*κ*B [41]. Regardless of the hypoxic induction, a significant LPS-PG induced activation of NF-*κ*B in PDL cells was seen even after 4 h. These findings point towards a more prominent role of LPS-PG in the activation of NF-*κ*B than hypoxia alone in PDL cells. Nevertheless, a synergistic effect on NF-*κ*B activation was observed after short term stimulation. Moreover, in clinically advanced disease stages with a more pronounced hypoxic microenvironment an enhanced activation and subsequent contribution of NF-*κ*B to the progression of inflammation is conceivable.

In line with this hypothesis we demonstrated a marked upregulation of NF-*κ*B staining in the gingiva and the periodontium of patients suffering from gingivitis or periodontitis. In gingivitis only keratinocytes, subepithelial fibroblasts, and some recruited immune cells were stained positively for NF-*κ*B. Instead, in periodontitis NF-*κ*B activation was seen widespread distributed in the tissue with intensive nuclear staining in endothelial and invaded immune cells reflecting the advanced stage of inflammation. The irreversible destructions seen in periodontitis may be caused by the excessive hypoxia- and pathogen-induced activation of NF-*κ*B followed by the expression of its respective target genes.

HIF is the key transcription factor for the adaptation of cells to hypoxic conditions and is predominantly regulated on the posttranslational level. However, LPS as well as inflammatory cytokines induces NF-*κ*B dependent the expression of the HIF-1*α* gene resulting in the expression of specific HIF target genes even under normoxic conditions [19, 42].

As a possible consequence of NF-*κ*B activation, we observed an induction of HIF-1*α* mRNA expression with a maximum after 4 h of LPS-PG incubation under hypoxic conditions. An induction of HIF-1*α* expression by LPS alone, as it was shown previously in monocytic cells could not be observed in PDL cells. This might be attributed to a differential and cell-type specific regulation of HIF-1*α* gene expression [19]. However, on the protein level LPS-PG, hypoxia and the combination of both induced an early and transient activation seen as nuclear translocation of HIF-1*α*. The most intense HIF-1*α* staining was observed after LPS-PG incubation under hypoxic conditions for 2 hours. As it was previously shown for human gingival cells [27], we were able to demonstrate the activation of HIF-1 by inflammatory mediators and hypoxia in PDL cells. In good agreement with these results immunostainings of periodontal tissues revealed an excessive HIF-1*α* accumulation in inflamed tissues when compared to healthy controls.

HIF and NF-*κ*B as well as their respective target genes are discussed to be associated with chronic inflammation, tumor induction, and progression. This was shown in cells origin from different organs. In particular HIF and HIF target genes were demonstrated to coincide with a malignant phenotype and bad prognosis [43, 44]. Apart from extracellular matrix degradation as discussed above the increase in vascular permeability facilitates the invasion of immune cells into the tissue and by this means promotes inflammation. VEGF is known to induce vascular permeability and angiogenesis [45]. Hypoxia, bacterial compounds, and several inflammatory cytokines have been shown to induce VEGF expression via activation of HIF-1 and NF-*κ*B [46–48]. Work from Artese et al. [49] and from Bletsa et al. [50] suggests that VEGF is an important factor in the pathogenesis of aggressive and chronic periodontitis. Therefore, we tested whether VEGF secretion was stimulated by LPS-PG and hypoxia in PDL cells. Only long-term stimulation with LPS-PG and hypoxia resulted in a significant increase of VEGF secretion. This was further supported by the immunohistochemical stainings of periodontal tissues, which depicted a substantial increase in VEGF positive cells distinctly distributed in inflamed tissue sections. While in tissue samples from healthy donors only keratinocytes were positively stained, in samples from patients suffering from gingivitis, VEGF staining could be assigned to keratinocytes, endothelial cells, and invading immune cells. The observation is in contrast to data published by Kasprzak et al. demonstrating an increased VEGF expression in tissue samples collected from patients with chronic periodontitis only in infiltrated immune cells [51]. Differences in classification of disease stages may explain these controversial results. In our tissue samples from patients with periodontitis we have seen an overall dramatic increase in the number of VEGF positive cells. Since we found in our* in vitro* experiments a significant VEGF release only after long-term incubation (>24 h) it is likely that elevated VEGF levels characterize a long existing chronic hypoxia and inflammation. The distinct VEGF upregulation in periodontitis may be of particular importance for the severe progression and exacerbation of periodontal diseases due to an increase in vascular permeability and a facilitated invasion of inflammatory immune cells.

## 5. Conclusion

Taken together, we demonstrated in the current study that hypoxia and LPS from* P. gingivalis* additively provoked the expression and release of MMP-1 and VEGF from PDL cells. Our findings underline the importance of PDL fibroblasts for the induction, progression, and persistence of gingivitis and severe periodontitis. HIF-1 and NF-*κ*B seem to be involved in these events. The secretion of matrix metalloproteinases and vascular permeability inducing factors facilitate the invasion of immune cells like monocytes/macrophages and neutrophils favoring the destruction of periodontal tissues.

## Figures and Tables

**Table 1:** Primer sequences used for real-time PCR.

Gene	Primer sequences (forward/reverse)
18S	fwd: CGGCTACCACATCCAAGGAA
	rev: GCTGGAATTACCGCGGCT

IL-1*β*	fwd: GTGGCCTTGGGCCTCAAGGA
	rev: TTGGGGAACTGGGCAGACTC

MMP-1	fwd: ACCCTCGCTGGGAGCAAACAC
	rev: AGTTGTCCCGATGATCTCCCCTGAC

HIF-1*α*	fwd: CTCCATTACCCACCGCTGAA
	rev: TCACTGGGACTATTAGGCTCAGGT
